# Lactoferrin Augmentation of the BCG Vaccine Leads to Increased Pulmonary Integrity

**DOI:** 10.1155/2011/835410

**Published:** 2011-04-28

**Authors:** Shen-An Hwang, Kerry J. Welsh, Marian L. Kruzel, Jeffrey K. Actor

**Affiliations:** ^1^Department of Pathology, University of Texas Medical School at Houston, 6431 Fannin, Houston, TX 77030, USA; ^2^Department of Integrative Biology and Pharmacology, University of Texas Medical School at Houston, 6431 Fannin, Houston, TX 77030, USA; ^3^Department of Pathology and Laboratory Medicine, University of Texas Medical School at Houston, MSB 2.214 6431 Fannin, Houston, TX 77030, USA

## Abstract

The goal of vaccination to prevent tuberculosis disease (TB) is to offer long-term protection to the individual and the community. In addition, the success of any protective TB vaccine should include the ability to limit cavitary formation and disease progression. The current BCG vaccine protects against disseminated TB disease in children by promoting development of antigenic-specific responses. However, its efficacy is limited in preventing postprimary pulmonary disease in adults that is responsible for the majority of disease and transmission. This paper illustrates the use of lactoferrin as an adjuvant to boost efficacy of the BCG vaccine to control organism growth and limit severe manifestation of pulmonary disease. This resulting limitation in pathology may ultimately, limit spread of bacilli and subsequent transmission of organisms between individuals. The current literature is reviewed, and data is presented to support molecular mechanisms underlying lactoferrin's utility as an adjuvant for the BCG vaccine.

## 1. Introduction

Tuberculosis (TB) is responsible for approximately 1.8 million deaths each year and is the leading bacterial cause of death worldwide [1]. Nearly one-third of the world is latently infected with *Mycobacterium tuberculosis* (MTB), making eradication of this disease extremely difficult. The current TB vaccine is an attenuated strain of *Mycobacterium bovis* Bacillus Calmette Guerin (BCG). BCG is effective in protecting against disseminated disease in children; however, its efficacy is limited in preventing pulmonary disease in adults [2–4]. While new vaccines are currently in development [5, 6], BCG remains the only TB vaccine approved for human use. Thus, one promising avenue is to develop adjuvants that are capable of improving efficacy of the existing BCG vaccine. This paper reviews the activity of one particular adjuvant, lactoferrin, focusing primarily on its immune modulatory effects and its potential to improve BCG effectiveness in the mouse model of TB infection. 

## 2. Host Immune Responses against MTB Infection

Vaccines represent one of the most powerful and cost-effective mechanisms for prevention of infectious disease, with many successful efforts leading to significant reduction in morbidity and mortality due to microbial assault [7]. The ultimate goal of vaccination to prevent TB disease encompasses not only long-term protection to the individual, but also to the community [8, 9]. The transmissibility of infection depends on its ability to escape from hosts that demonstrate adequate immunity. A goal of a protective TB vaccine should include the ability to limit cavity formation, which is critical to subsequent spread of disease [10, 11]. Many scientists believe that the key to a successful TB vaccine includes generation of responses that limit deleterious pathology in the lung. Alteration of the immunopathological consequences of mycobacterial infection may lead to subsequent reduction in transmission of human disease. 

The generation of a T_H_1 immune response is critical for host control of mycobacteria [12]. Infection with MTB begins with exponential growth of bacilli in macrophages. A nascent granuloma forms as a result of the accumulation of infected and noninfected macrophages responding to proinflammatory biomediators, likely triggered by mycobacterial-derived glycolipid factors [13–17]. Dendritic cell presentation of TB antigens, in the presence of cytokines such as IL-12, initiates a CD4^+^  T_H_1 immune response [18]. IFN-*γ* production by T_H_1 cells activates macrophages, resulting in phagosome acidification and production of both reactive oxygen and nitrogen species capable of killing MTB [12]. CD4^+^ T-cells also assist in the development of CD8^+^ cytotoxic T-cells, which are critical for control of disease pathology [12]. The mechanisms underlying CD8^+^ stimulation towards generation of specific responses towards MTB antigens are under active investigation. Finally, recent studies suggest that T_H_17 cells, a relatively newly defined T-helper cell subset with modulatory functions, may also play an important role in protection against MTB in vaccinated animals [19]. Therefore, immunomodulatory agents used as adjuvants to enhance efficacy of the BCG vaccine are expected to produce not only strong T_H_1 responses, but solid cytotoxic and regulatory responses as well. 

MTB subverts immune recognition within macrophages and limits phagosome-lysosome fusion events necessary for destruction of organisms [21–23] and subsequent development of adaptive responses [24, 25]. The BCG vaccine generates host protective responses against MTB infection by promoting development of a mycobacterial antigen-specific delayed type hypersensitivity (DTH), specifically a T-cell helper type-1 (T_H_1) immunity with antigen-specific production of interferon-gamma (IFN-*γ*) [26]. In turn, these T-cell responses activate macrophages, allowing containment and control of organism growth. The strong T_H_1 immunity is in part counterregulated by T_H_2 elicited cytokines [27, 28]. Thus, an effective TB vaccine requires induction of strong T_H_1 immunity, emphasizing the role of adjuvants that can skew T-cell differentiation as an important component of rational vaccine design [28–30]. Many investigators speculate that use of defined adjuvants to alter development of specific memory T-cell subsets would function more effectively over longer periods to combat TB infection [31]. 

New evidence identified lactoferrin as a regulator of immune responses to a variety of infectious and injurious stimuli. Lactoferrin is a member of the transferrin family and is found in mucosal secretions as well as neutrophil granules [32]. The neutrophilic glycoform of lactoferrin plays a critical role in immune modulation [32]. Serum lactoferrin concentration is normally low, at less than 1 *μ*g/mL, but increases considerably during inflammation and injury [20, 33]. Receptors for lactoferrin are found on many immune cells, including dendritic cells, macrophages, and T-cells [34–36], contributing to the wide range of reported immunomodulatory properties. These include activation of macrophages, increasing polymorphonuclear cell phagocytosis, promotion of B- and T-cell maturation, and enhancement of antigen-specific immune responses [37–39]. 

## 3. Lactoferrin Modulation of Innate Immunity

Lactoferrin has a number of effects on innate antigen presenting cells (APCs) that have the potential to modulate T-cell responses. APCs, such as dendritic cells, present antigen to naive CD4^+^ T-cells via major histocompatibility complex II (MHC II) and costimulatory molecules such as CD80, CD86, and CD40 [40, 41]. IL-12 production by APCs promotes development of naive CD4^+^ T-cells to the T_H_1 phenotype [42, 43]. Thus, modulation of APC surface molecule expression and cytokine production may allow enhancement of the protective immune response against MTB infection. 

Lactoferrin has the potential to enhance macrophage and dendritic cell function as antigen presenters to activate CD4^+^ T-cells. IFN-*γ*-stimulated macrophages infected with mycobacteria, including BCG have decreased expression of MHC II [44–46]. Addition of lactoferrin to activated macrophages infected with BCG significantly enhanced MHC II expression [47, 48]. The CD86 : CD80 ratio was increased in macrophages and dendritic cells infected with BCG and stimulated with lactoferrin [47, 49], suggesting that lactoferrin-treated APCs are better able to promote T-cell activation during antigen presentation by infected cells [50, 51]. Indeed, BCG-infected macrophages and dendritic cells cultured in the presence of lactoferrin significantly increased IFN-*γ* production from CD3^+^ and CD4^+^ cells compared to APCs cultured without lactoferrin [47, 49]. Furthermore, lactoferrin enhanced expression of CD40 on peritoneal macrophages and on the mouse macrophage cell line RAW 264.7 [52]. Human immature dendritic cells incubated with recombinant human lactoferrin increased CD80, CD86, CD83, human leukocyte antigen II, as well as chemokine receptors involved in migration to draining lymph nodes [53]. 

As a proof of concept, we demonstrated that lactoferrin could alter production of inflammatory cytokines from LPS stimulated murine-derived or human-derived macrophages, mimicking in part the status of infected cells. We initially reported that lactoferrin was effective at augmenting proinflammatory responses from stimulated splenocytes and macrophages [54, 55]. For example, when whole splenocytes isolated from C57BL/6 mice were stimulated with low levels of LPS (100 ng/mL), TNF-*α*, and IL-6 were modulated by increasing concentrations of lactoferrin (1, 10 *μ*g/mL). In addition, lactoferrin was able to directly stimulate TNF-*α*, IL-6, and IL-12 production from J774A.1 and RAW 264.7 macrophages [54, 55]. A direct comparison of novel recombinant human neutrophilic lactoferrin [56] with milk-derived lactoferrin was performed. The neutrophilic form was able to induce high IL-12 production from the cultured cells. For example, human THP-1 cells stimulated with LPS incubated in the presence of increasing concentrations of bovine lactoferrin, human milk-derived, or recombinant human neutrophilic lactoferrin all significantly diminished TNF-*α* production in a dose-dependent manner. However, only the human neutrophilic lactoferrin and the bovine-derived lactoferrin were able to significantly alter IL-6 and IL-12p40 production. The milk lactoferrin isoform, which contains fucose, was not able to do so. Nonfucosylated moieties are characteristic of human neutrophilic leucocytes whereas human milk-derived LF contains fucose residues at the *N*-acetylglucosamine residue [57]. While others have demonstrated that *N*-acetylneuraminic (sialic) acid as a terminal sugar is important in propagation of other immune responses, it was not a factor in modulation of activated macrophages. Overall, the results for the recombinant human lactoferrin were nearly identical to those obtained using the bovine-derived lactoferrin.

IL-12 is an essential modulator of the T_H_1 cytokine IFN-*γ*, both in the induction of T_H_1 cells from naive T-cells and in maximizing IFN-*γ* production from differentiated T_H_1 effector and memory cells [58, 59]. Lactoferrin was shown to function on leukocytes *in vivo*; intraperitoneal injection of lactoferrin into mice increased IL-12 production from recovered peritoneal macrophages [37]. Others have demonstrated that oral administration of lactoferrin increases IL-12p40 expression that is accompanied by a decrease in IL-10 expression in the small intestines [60]. Indeed, in the presence of lactoferrin, macrophages infected with BCG clearly demonstrated significant increased ratio of IL-12 relative to IL-10, a cytokine that negatively impacts IL-12 [37]. Additionally, lactoferrin increased production of TGF-*β*1 from BCG-infected dendritic cells and macrophages [47, 49]. TGF-*β*1 in the presence of IL-6 has the potential to promote the development of T_H_17 responses [61], which have been shown to play an important role in the generation of memory and recall responses to MTB antigens [19, 62]. Taken together, these studies indicate that lactoferrin is a strong modulator of APC function. The effect of lactoferrin on innate cells involved in the initial encounter with microbes gives it the potential to enhance the development of acquired immunity, with clear molecular mechanisms to further support its use as a vaccine adjuvant. 

## 4. Lactoferrin Modulation of Adaptive Immune Responses

**Figure 1:**
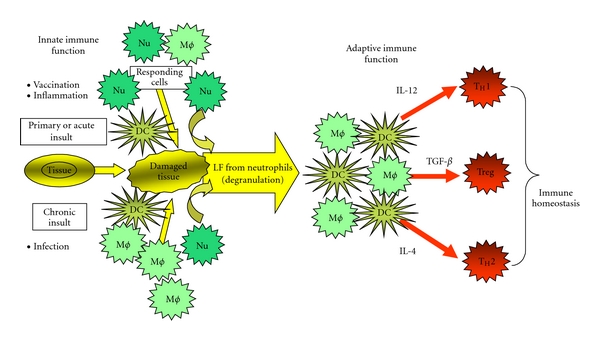
Lactoferrin as a bridge between innate and adaptive immune function. Insult, defined as infection or inflammatory stimulation, leads to activation of the monocyte/macrophage system (MØ), including neutrophils (Nu). In the case of primary vaccination, dendritic cells (DCs) are also directly mediated. Activated neutrophils (Nu) degranulate at the site of inflammation and release lactoferrin. Depending on the magnitude and/or duration of the insult, DCs mature to express differential amounts of specific cytokines that affect local environments to subsequently promote T-cell polarization into T_H_1, T_reg_, or T_H_2 phenotypic populations (diagram adapted from [20]).

Lactoferrin is a modulator capable of bridging innate and adaptive immune functions (Figure 1). Soluble products released during innate reactivity (whether due to infection, immunization, or insult) serve to direct adaptive responses. Lactoferrin also has the potential to limit insult-induced oxidative stress while at the same time directing DCs to promote T-cell polarization [20, 32]. Receptors for lactoferrin are found on CD4, CD8, and *γδ* T-cells [63]. Lactoferrin also affects the level of costimulatory surface molecules that modulate T-cell activities, indicating that lactoferrin may affect T-cell activity and response to antigen. Human lactoferrin promotes the maturation of double negative mouse T-cells preferentially towards CD4 T-cells, possibly by activating the MAP kinase pathway through Erk2 and p56^lck^ [64, 65]. Oral lactoferrin administration to mice increased total circulating granulocytes as well as CD4 and *γδ* T-cells [60]. Expression of leukocyte function associated antigen, an adhesion molecule involved in cell-to-cell contact on both CD4^+^ and CD8^+^ T-cells, was increased by lactoferrin [66]. Lactoferrin increased expression of the human T-cell *ζ*-chain that is a component of the CD3 T-cell receptor complex involved in signaling [67]. 

Lactoferrin promotes polarization of naive T-cells to either T_H_1 or T_H_2 phenotypes depending on the antigen and cytokine milieu. Classical studies demonstrated that lactoferrin can promote the production of cytokines necessary for the development of a T_H_1 response, with proven enhancement of the DTH response to ovalbumin, sheep red blood cells, and BCG [37, 68, 69]. The mechanisms appear to be unique relative to effects on mature T cells, where differential activities with regard to effector functions of T cells with antigen specificity were found [70]. Transgenic mice expressing human lactoferrin demonstrated increased IFN-*γ* and TNF-*α*, accompanied by decreased IL-10 and IL-5, during infection with *Staphylococcus aureus* [71]. Lactoferrin administered orally increased T_H_1 T-cell responses, measured by increased levels of IFN-*γ*, in naive and tumor-harboring mice [72, 73]. Lactoferrin increased the IL-12 : IL-10 ratio in LPS stimulated splenocytes [74]. Additionally, elimination of chronic hepatitis C virus is enhanced by the addition of lactoferrin to interferon therapy, possibly by enhancing T_H_1 responses [75]. Conversely, lactoferrin decreased IFN-*γ* and increased IL-10 in an infection model of *Toxoplasma gondii*, suggesting a promotion of a T_H_2 response [76]. The effects of lactoferrin on the newly defined T-cell subset, T_H_17 cells, are currently unknown; however, preliminary studies indicate that lactoferrin may promote IL-17 responses to mycobacterial antigens (Hwang SA and Welsh KJ, unpublished data). 

Lactoferrin also modulates B-cell responses. Incubation of immature B-cells with lactoferrin enhanced their ability to promote proliferation of antigen-specific T-cells [39]. Additionally, lactoferrin promotes the maturation of mouse immature B-cells as measured by increased expression of IgD and the complement 3 receptor [39]. Lactoferrin demonstrated an increase in the production of IgG and IgA from Peyer's patches in mice [77]. Antibodies in mice treated with lactoferrin had increased levels of IgG in both the intestine and serum [78]. Furthermore, lactoferrin overcomes the suppressive effects of cyclophosphamide and methotrexate by increasing the number of antibody-forming cells and humoral responses to sheep red blood cells [56, 79, 80]. These studies indicate that lactoferrin has direct effects on B-cells and potentially modulates their function as APCs to promote T-cell responses. 

## 5. Lactoferrin as Vaccine Adjuvant

Lactoferrin is an excellent candidate for a vaccine adjuvant due to its effects on APCs and modulation of the adaptive immune response. Lactoferrin may specifically enhance the effectiveness of vaccines due to its proven enhancement of the specific immune reactions to defined antigens, including BCG [37, 68, 69]. Most critical, lactoferrin has been shown to protect against immune-mediated tissue damage [81, 82]. We hypothesize that the effect of lactoferrin is directly via host immune modulation, as it has been shown that lactoferrin has no direct microbicidal activity on BCG, whether grown in broth culture or when added to monocytes already infected with organisms [47, 49].

Studies from our laboratory demonstrate that bovine lactoferrin, as well as human lactoferrin, added to the BCG vaccine led to better protection against challenge with virulent organisms, indicated by decreased bacterial burden in the lung and spleen, than BCG alone [55, 81, 83, 84]. Mice vaccinated with BCG/bovine lactoferrin had increased lung expression of IFN-*γ* mRNA at early times postchallenge with virulent MTB, suggesting enhanced T_H_1 responses at sites of clinical importance. Splenic recall responses to heat-killed BCG in mice given the BCG/bovine lactoferrin vaccine demonstrated increased levels of IFN-*γ* and other proinflammatory mediators, compared to mice vaccinated with only BCG. IL-4 was reduced in these groups [81]. Lung histopathology was also significantly reduced in mice immunized with BCG/lactoferrin, demonstrating focal, lymphocytic, granulomas surrounded by normal lung parenchyma. The enhanced protective effects of BCG/bovine lactoferrin vaccine extend to BALB/c mice, which typically demonstrate decreased T_H_1 responses to MTB compared to C57BL/6 mice [84]. 

**Figure 2:**
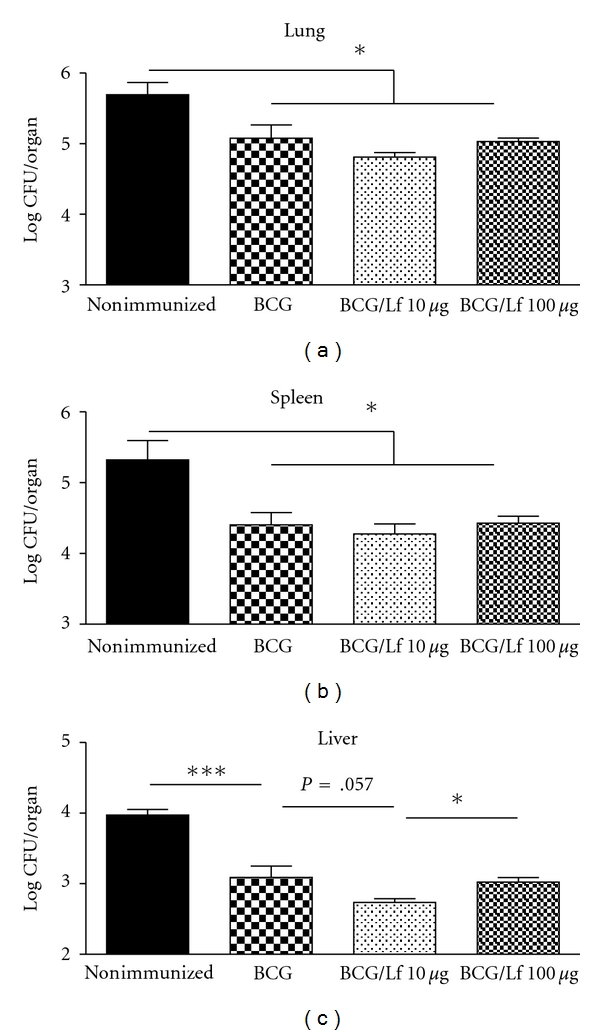
Reduced bacterial loads in BCG-immunized mice after infectious challenge with MTB. C57BL/6 mice were immunized with BCG (1 × 10^6^ CFU/mouse; Pasteur strain TMC 1011, ATCC, Manassas, VA) or BCG/bovine lactoferrin (10 or 100 *μ*g/mouse) and boosted at 8 weeks. One group remained nonimmunized. At 12 weeks postboost, mice were aerosol infected with a low dose (approximately 100 CFU/mouse) Erdman MTB (TMC 107, ATCC) and monitored through day 65 postinfection for organ bacterial load. All vaccinated mice were able to reduce bacterial load in tissue following infectious challenge. Minimum number of animals per group was 6 for controls and 10 for immunized mice. *P < .05; ***P < .001.

Recently completed experiments identified lactoferrin to function as an adjuvant at lower doses than originally reported [55, 83], thus making this a more attractive adjuvant for clinical use. Specifically, C57BL/6 mice were immunized with BCG or with BCG and bovine lactoferrin at 10 or 100 *μ*g/mouse. Mice were boosted at 8 weeks. At 12 weeks postboost, mice were aerosol infected with MTB strain Erdman (TMC 107, ATCC) and monitored through day 65 postinfection. One group remained nonimmunized. Generally, all mice immunized with BCG or BCG/lactoferrin demonstrated a significant decrease in lung, spleen, and liver organ colony forming units (CFUs) compared to the nonimmunized group. In liver, mice immunized with BCG/lactoferrin (10 *μ*g) demonstrated slightly greater decrease in organ bacterial loads compared to the BCG only and BCG/lactoferrin (100 *μ*g) groups (Figure 2). Previous studies indicated that the 100 *μ*g dose of lactoferrin was able to reduce organ bacterial loads at 4 weeks postchallenge [55, 84], but this was evident at earlier times postchallenge than reported here. The fact that lactoferrin may be used at doses tenfold lower than previously reported confirms its ability to function as an immune mediator at low levels and has been reported for its use in other models of inflammation [20, 33]. 

**Figure 3:**
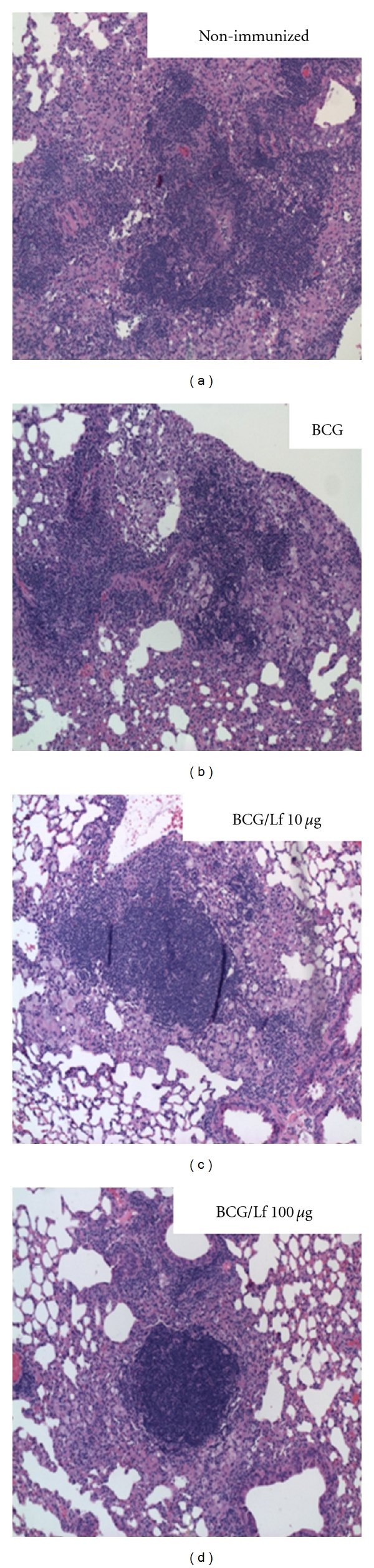
Protective histopathology following infectious challenge in lactoferrin adjuvant immunized mice. C57BL/6 mice were immunized as detailed in Figure 2 with BCG or BCG/bovine lactoferrin (10 or 100 *μ*g/mouse) and aerosol challenged 12 weeks after final boost. At day 65 postchallenge, lungs were collected, formalin fixed, and stained with hematoxylin and eosin (H&E). Comparison is made to nonimmunized, infected controls. A mixed presence of lymphocytes and activated macrophages indicative of a protective response is identified in the BCG alone vaccinated group. The addition of lactoferrin at either concentration resulted in a confined granulomatous response with focal lymphocytic accumulation and limited to no aggravated macrophage or polymorphonuclear insult. Images were visualized at 100x magnification.

**Figure 4:**
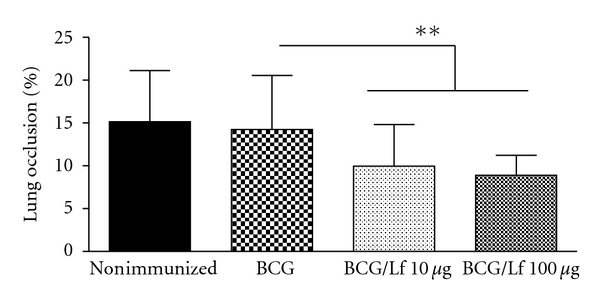
Quantitative analysis of histopathological protective response due to lactoferrin vaccination. Quantitative assessment of BCG immunization with or without lactoferrin (at 10 or 100 *μ*g/mouse) as described in Figure 2 revealed significant reduction in lung occlusion following aerosol infection with virulent mycobacteria at 65 days postchallenge. Quantitation of lung occlusion percent was completed using Image J (NIH) by comparing total section area with total area of granulomas. Number of animals per group ranged from 6 controls to 10 in the vaccinated groups. **P < .01; NS: no significant difference between groups.

Histological analysis revealed that immunization with BCG and lactoferrin at either dose resulted in similar improvements in pathological development to infectious challenge. The addition of lactoferrin to the vaccine led to focal granulomas with tight lymphocytic clusters and minimized inflammation in the surrounding parenchyma. In contrast, the nonimmunized and BCG immunized groups developed large granulomas with loosely clustered lymphocytes and activated foamy macrophages, along with manifestations of inflammation in the surrounding tissue (Figure 3). The BCG alone group demonstrated histological changes, but they included the presence of activated macrophages, which are assumed to contribute to the production of deleterious and pathologically active mediators [85–88]. Quantitative analysis of the percentage of lung tissue occupied by granuloma lesions reconfirmed the histological findings (Figure 4). Mice vaccinated with BCG admixed with 10 or 100 *μ*g/mouse of lactoferrin showed significant decreases in lung occlusion percent compared to both the BCG only and nonimmunized groups. Of interest was the observation that large pathological protection was apparent in the absence of reduction in bacterial loads and that this reduction in pathology was seen using lower levels of lactoferrin. In all studies specific biomarkers reflected the activity of a disease process (bacterial loads, granuloma formation, or production of cytokines). While these indicators should theoretically correlate (either directly or inversely) with disease progression, in practice many biomarkers are likely dependent upon elaborate mechanisms. As lactoferrin is a multifunctional protein, it activates multiple pathways with differential modifications to specific disease processes.

While the bovine form of lactoferrin added to the BCG vaccine enhanced the effectiveness of BCG vaccination, it is unlikely that bovine lactoferrin can be used parenterally in humans due to the possibility of inducing allergic responses. Human recombinant lactoferrin that has identical glycosylation patterns to neutrophilic lactoferrin was recently developed using a *Pichia pastoris* expression system [56]. The sialylated and nonsialylated variants of human lactoferrin were admixed into the BCG vaccine and examined for their ability to provide enhanced protection following challenge with virulent MTB [81]. The sialylated form of recombinant human lactoferrin added to the BCG vaccine enhanced protection, as indicated by decreased bacterial load in the lung, spleen, and liver. Antigen-specific recall responses to heat-killed BCG demonstrated increased IFN-*γ* by splenocytes of the mice vaccinated with sialylated human lactoferrin as an adjuvant. Lung inflammatory pathology was also significantly reduced by the addition of recombinant human lactoferrin.

The reduction of lung pathology by addition of lactoferrin to the BCG vaccine has a number of significant implications. Destruction of lung tissue likely contributes to the transmission of MTB and is an important cause of morbidity [89]. Indeed, a strategy for rational vaccine design includes mechanisms that reduce immunopathology and dissemination of infection at later time points [8, 9]. Vaccines that reduce pathology after challenge with virulent MTB may also correlate with disease protection [31]. 

## 6. Summary

Our underlying hypothesis for utilizing lactoferrin to boost efficacy of the BCG vaccine is in agreement with assessment that prolonged survival may be predicated on changes in the pathological manifestation of disease within lung tissue [90], with improvement seen in the absence of decreased bacillary load. Indeed, limiting pathology would create a “firebreak” to slow transmission, even in spite of organisms remaining hidden in various organs [91]. Evidence to support this is found in the guinea pig model, suggesting that survival following challenge may occur in vaccinated animals in the absence of decreased early bacillary loads [11, 92, 93]. The data presented in this paper indicates that lactoferrin given at 10 *μ*g/mouse can function with resultant protective pathology while retaining full adjuvant activity [56, 81, 83]. The ability to lower the dose of lactoferrin while maintaining activity is necessary for clinical utility of lactoferrin as an adjuvant to boost BCG vaccine in humans.
